# Survival outcomes in EIF2AK4 mutation-associated pulmonary arterial hypertension: seeking clarity in contrast

**DOI:** 10.1093/ehjcr/ytae538

**Published:** 2024-10-07

**Authors:** Jennie Han, Zehra Kadani, Laura C Price, Aleksander Kempny, Bhavin Rawal, Stephen J Wort, Colm McCabe

**Affiliations:** National Pulmonary Hypertension Centre, Royal Brompton Hospital, Part of GSTT Foundation Trust, Sydney Street, London SW3 6NP, UK; National Pulmonary Hypertension Centre, Royal Brompton Hospital, Part of GSTT Foundation Trust, Sydney Street, London SW3 6NP, UK; National Pulmonary Hypertension Centre, Royal Brompton Hospital, Part of GSTT Foundation Trust, Sydney Street, London SW3 6NP, UK; National Heart and Lung Institute, Imperial College, London SW7 2AZ, UK; National Pulmonary Hypertension Centre, Royal Brompton Hospital, Part of GSTT Foundation Trust, Sydney Street, London SW3 6NP, UK; National Heart and Lung Institute, Imperial College, London SW7 2AZ, UK; National Pulmonary Hypertension Centre, Royal Brompton Hospital, Part of GSTT Foundation Trust, Sydney Street, London SW3 6NP, UK; National Pulmonary Hypertension Centre, Royal Brompton Hospital, Part of GSTT Foundation Trust, Sydney Street, London SW3 6NP, UK; National Heart and Lung Institute, Imperial College, London SW7 2AZ, UK; National Pulmonary Hypertension Centre, Royal Brompton Hospital, Part of GSTT Foundation Trust, Sydney Street, London SW3 6NP, UK; National Heart and Lung Institute, Imperial College, London SW7 2AZ, UK

**Keywords:** Case report, Pulmonary arterial hypertension, Exercise, Intra pulmonary shunt, Right ventricle

## Abstract

**Background:**

Pulmonary veno-occlusive disease (PVOD) is a rare cause of pulmonary arterial hypertension (PAH) characterized by widespread fibrous intimal proliferation of pre-septal pulmonary venules and a lower lung diffusion capacity for carbon monoxide when compared to classical PAH. Mutations in the eukaryotic translation initiation factor 2 alpha kinase 4 (EIF2AK4) gene have been linked to the development of PVOD, with the worst prognosis seen in homozygous mutation carriers.

**Case summary:**

We describe two patients with homozygous EIF2AK4-associated PVOD, who despite typical clinical features at presentation have demonstrated a remarkable response to pulmonary vasodilator therapy and comparatively benign clinical courses. Intrapulmonary shunt (IPS) was evident on resting contrast transthoracic echocardiography (CTTE) in both patients undertaken 4 and 36 months following diagnosis. At 2 and 10 years of follow-up, respectively, both patients retain preserved right heart function and remain in the World Health Organization functional class II. This case series contrasts strikingly with prior reports of patients with classical PAH where IPS that develops in response to pulmonary vasodilator treatment has been associated with dramatic reduction in systemic oxygen saturations, necessitating withdrawal of therapy.

**Discussion:**

In two patients with PVOD associated with homozygous EIF2AK4 mutations, IPS may act to offload the right ventricle with relative preservation of systemic exercise saturations and a more favourable prognosis. Greater use of CTTE in patients with PVOD as well as PAH with lower lung diffusion capacity may lend insight into the clinical and prognostic relevance of IPS in these patient subgroups with otherwise poor prognosis.

**Learning points**

- Mutations in the EIF2AK4 gene are associated with both sporadic and familial pulmonary veno-occlusive disease (PVOD), a rare form of pulmonary arterial hypertension (PAH).
- Two patients with biallelic EIF2AK4-associated PVOD demonstrate favourable prognoses, both with evidence of intrapulmonary shunt on contrast transthoracic echocardiogram.
- Intrapulmonary shunt may help reduce pulmonary vascular load and warrants wider evaluation in PAH populations with low DLCO.

## Introduction

Pulmonary arterial hypertension (PAH) has witnessed important recent advances in molecular pathophenotyping following the genomic sequencing of familial clusters and large-scale disease cohorts. Among the pathological sequence variants associated with PAH, mutations in the eukaryotic translation initiation factor 2 alpha kinase 4 (*EIF2AK4*) gene, in both homozygous or compound heterozygote state, are now established in the diagnostic algorithm of both heritable and sporadic pulmonary veno-occlusive disease (PVOD), a condition characterized by fibrous intimal proliferation of pulmonary septal veins and pre-septal venules recently redefined in ESC/ERS guidelines as PAH with features of venous/capillary (PVOD/PCH) involvement.^1^

*EIF2AK4* is a serine/threonine protein kinase that phosphorylates the α subunit of eukaryotic initiation factor 2 and activates the integrated stress response pathway inducing changes in gene expression in response to amino acid deprivation.^2^ Although a pathophysiological mechanism between loss-of-function *EIF2AK4* mutations with pulmonary vascular cell proliferation and remodelling remains unclear, patients with biallelic *EIF2AK4* mutations are recognized for their younger age at presentation (median age 29 vs. 51 years in patients with other PAH-associated mutations), lower diffusion capacity for carbon monoxide (*D*_LCO_) (typically <50% of predicted), and less favourable response to pulmonary vasodilator therapies compared to other PAH patient subgroups.^3^ In *EIF2AK4*-associated PAH, the clinical role of genetic testing is further underscored by potentially earlier patient identification as well as the lack of reliability using radiological features on thoracic computed tomography (CT) to distinguish these patients from those with idiopathic PAH.

## Summary figure

**Figure:**
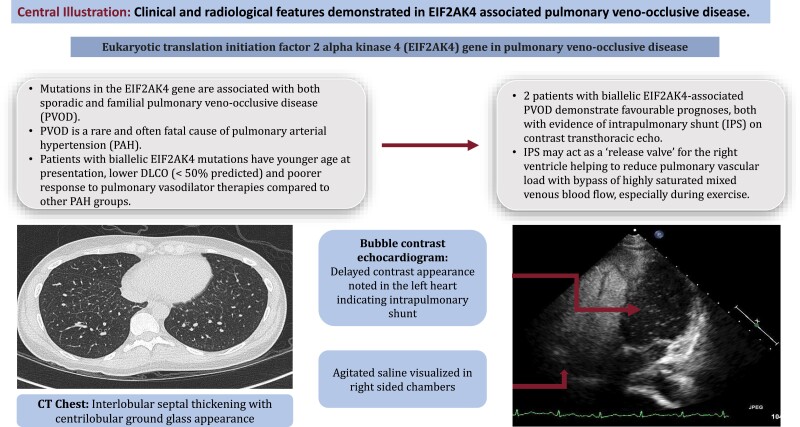


## Patient 1

**Figure 1:**
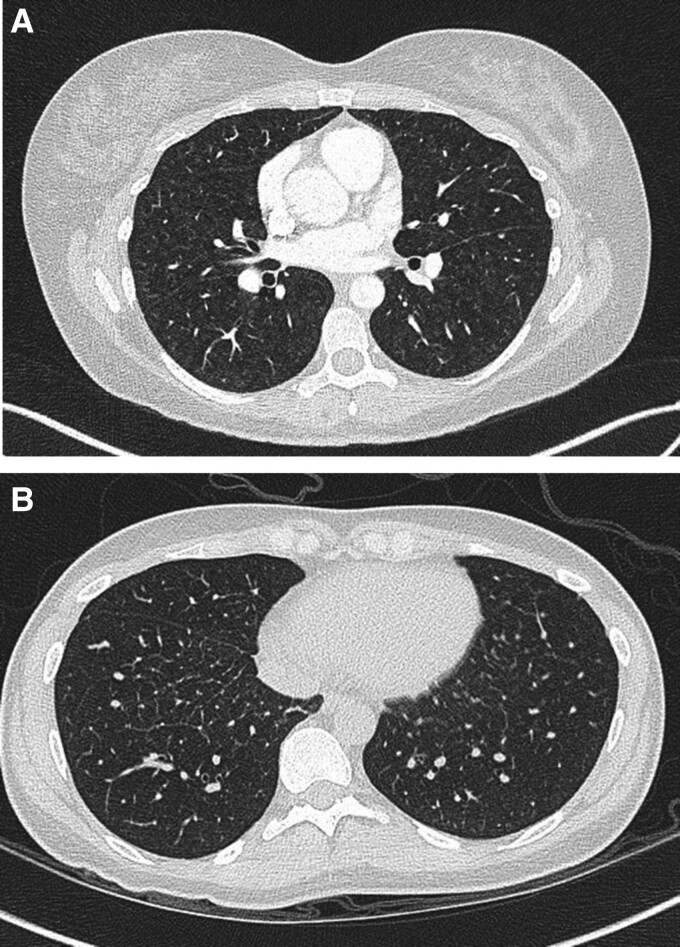
(*A*) High-resolution computed tomography of Patient 1 at diagnosis showing soft ground glass nodularity and subtle interlobular septal lines. (*B*) High-resolution computed tomography of Patient 2 at follow-up showing interlobular septal thickening and mild parenchymal lung nodularity.

A 40-year-old woman presented with a 3-year history of progressive shortness of breath and pre-syncopal symptoms on exertion following the birth of her son. Her family history included consanguine marriage in her grandparent lineage. Cardiorespiratory examination was unremarkable with resting oxygen saturations of 96% on FiO2 21%. Echocardiogram revealed a moderate to severely dilated right ventricle (RV) with moderately reduced systolic function, an estimated pulmonary artery systolic pressure (PASP) of 56 mmHg, fractional area change (FAC) 25%, and inter-ventricular septal flattening consistent with RV pressure and volume overload. Pulmonary function tests showed normal lung volumes with severe reduction in gas transfer (TLco 37%, Kco 40%). She underwent right heart catheterization which showed pre-capillary pulmonary hypertension with no response to inhaled nitric oxide (*Table 1*, Patient 1). A CT chest demonstrated enlarged right heart chambers and centrilobular ground glass opacification in lung parenchyma (*Figure 1A*). She was commenced on pulmonary vasodilator therapies, tadalafil and ambrisentan, both of which were up-titrated over several weeks, and genetic screening was obtained.

**Table 1:** Clinical characteristics of two patients with biallelic EIF2AK4 mutation-associated PAH

	Patient 1 (40 female)	Patient 2 (34 female)
Genetic mutation	Homozygous pathogenic missense variant EIF2AK4: c,1849G>C, p.(Ala617Pro)	Homozygous pathogenic EIF2AK4, variant details unavailable
Follow-up duration	1 year and 6 months	8 years and 3 months
Digital clubbing	Absent	Absent
Echocardiogram at diagnosis		
RV systolic function	Moderately reduced	Mildly reduced
FAC (%)	25	27
RV morphology	Moderate-severely dilated	Mildly dilated
TR *V*_max_ (m/s)	3.50	Unable to quantify
PASP (mmHg)	56 mmHg	Unable to quantify
ESC probability of PAH	High	Intermediate
Cardiac MRI at diagnosis		
LVEF (%)	64	61
RVEF (%)	58	51
RV EDV (mL)	176	108
RV ESV (mL)	75	53
RV SV (mL)	101	55
Gadolinium enhancement	Late enhancement in RV septal insertion	No significant early/late
Haemodynamics at diagnosis		
PAP (S/D/M) (mmHg)	60/21/39	61/27/41
PCWP (mmHg)	2	6
CO (L/min)	4.15	4.67
PVR (WU)	8.9	7.5
Nitric oxide vasoreactivity testing	No response	No response
Haemodynamics at 12-month follow-up		
PAP (mmHg)	52/24/34	32/16/24
PCWP (mmHg)	8	12
CO (L/min)	9.4	5.6
PVR (WU)	2.8	2.1
Pulmonary vasodilator treatment	Tadalafil, ambrisentan	Sildenafil, ambrisentan
Contrast echocardiogram findings	4 months after presentation; moderate right-to-left shunt, late contrast in LA	3 years after presentation; moderate right-to-left shunt from left pulmonary veins
CT findings	Centrilobular ground glass opacification	Subtle interlobular septal thickening and small calcified lung nodules
Lung function (at diagnosis)		
FEV_1_ (%)	108	93
FVC (%)	117	100
DLco (%)	37	24
Kco (%)	40	30
Cardiopulmonary exercise testing data (on pulmonary vasodilator treatment)		
Peak VO_2_ (%)	53	34
VE/VCO_2_ slope	55	56
Peak O_2_ sats (%)	90	94
Rest A—a gradient (kPa)	5.3	–
Peak A—a gradient (kPa)	9.8	–
Rest Vd/Vt (%)	19	–
Peak Vd/Vt (%)	32	–
Lactate (mmol/L)	2.9	3.1

PAP, pulmonary artery pressure; S/D/M, systolic/diastolic/mean; PCWP, pulmonary capillary wedge pressure; CO, cardiac output; PVR, pulmonary vascular resistance; FEV_1_, forced expiratory volume in 1 s; FVC, forced vital capacity; TLco, diffusion capacity for carbon monoxide; Kco, diffusion capacity for carbon monoxide/alveolar ventilation.

**Figure 2:**
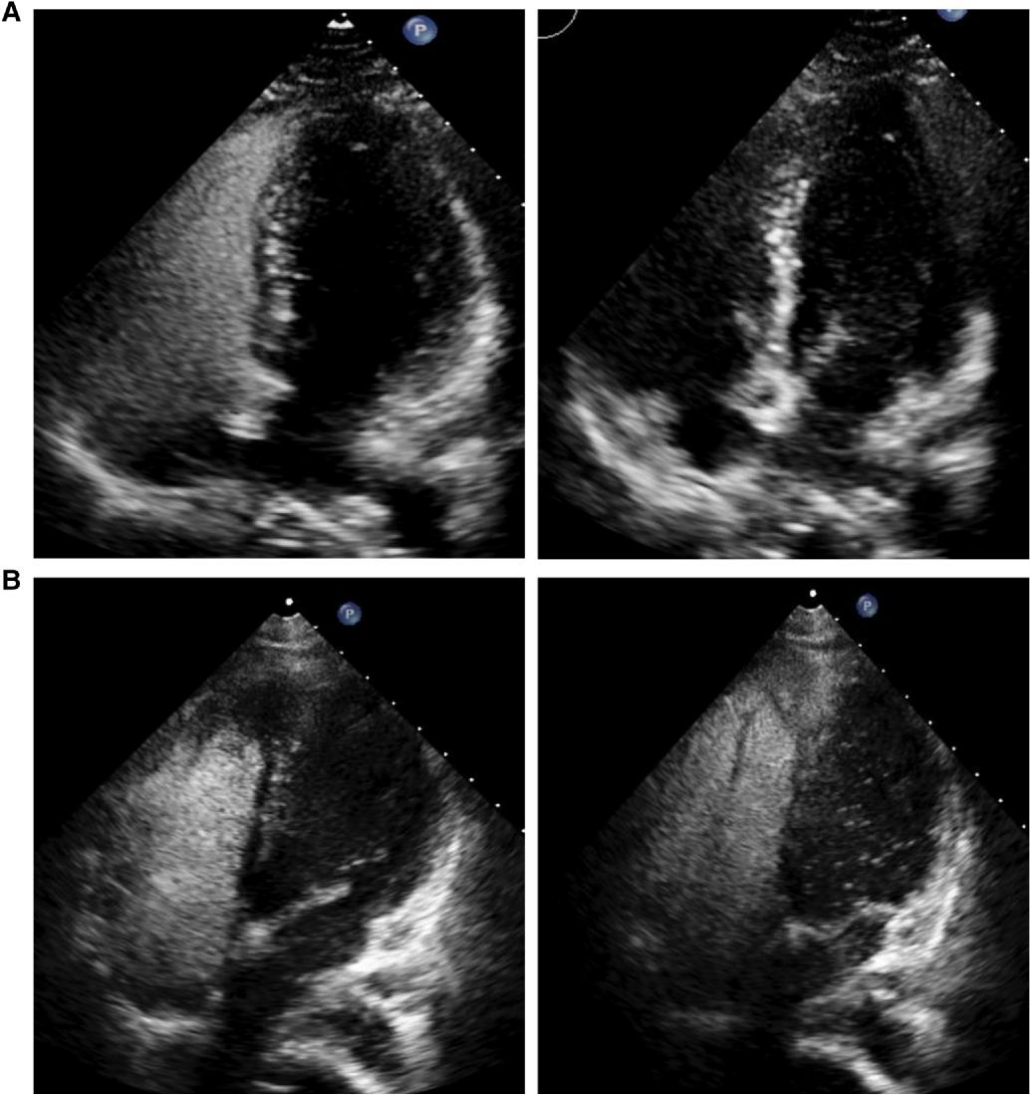
Contrast transthoracic echocardiography in Patient 1 (top left, top right images) and Patient 2 (bottom left, bottom right) showing late appearance of bubbles in left ventricle following peripheral vein injection.

**Figure 3:**
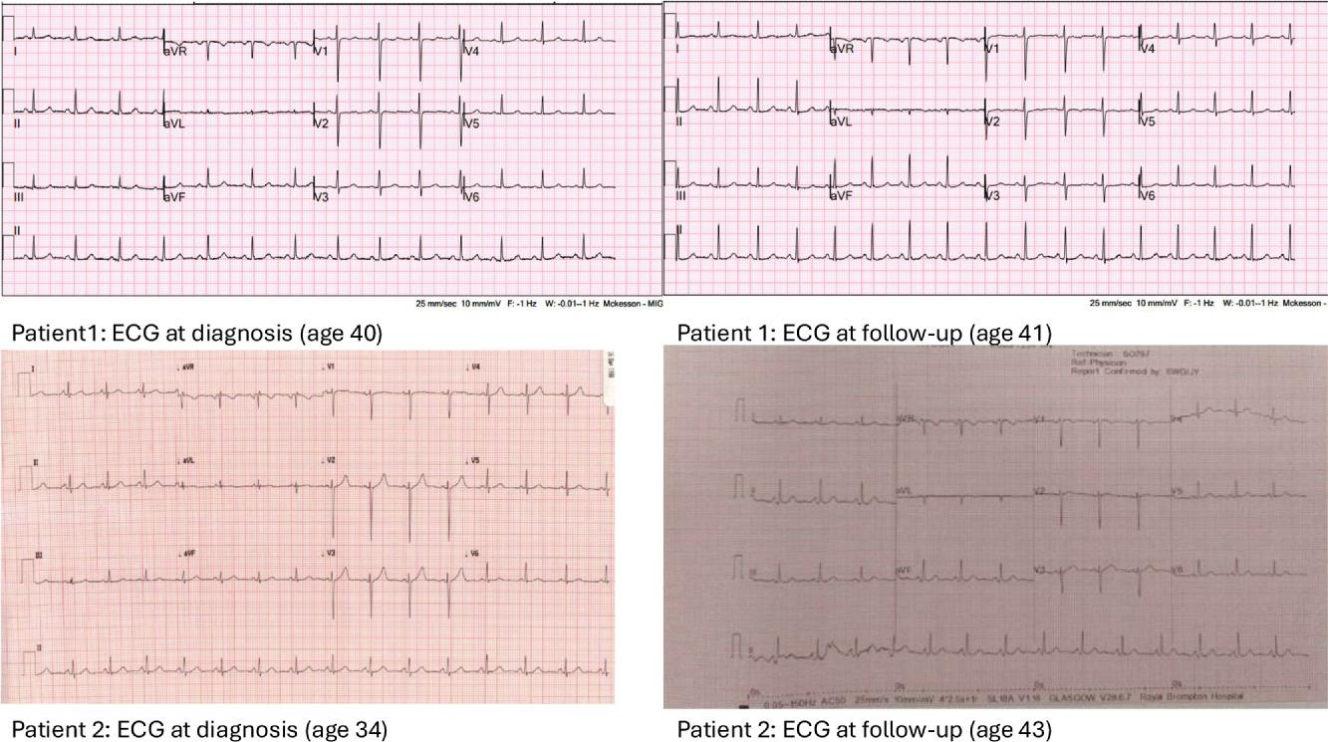
Twelve-lead ECGs for both patients at presentation and follow-up.

After 2 months of therapy, there was improvement in the patient’s subjective exercise tolerance. Genetic testing identified a novel homozygous mutation in the EIF2AK gene, and further genetic screening of the family members was advised, which identified a similar mutation in a younger sibling. An echocardiogram showed a mildly dilated RV, low normal systolic function, normal tricuspid annular plane systolic excursion, and FAC 36.6%. Given evidence of low normal resting saturations 94%, a bubble study was requested which confirmed moderate right-to-left shunting with delayed appearance of contrast in the LA arising from pulmonary veins (*Figure 2A*). Repeat right heart catheterization showed improvement in pulmonary haemodynamics (*Table 1*, Patient 1). Follow-up lung function tests also demonstrated a small increase in TLco. A resting ECG showed maintenance of normal sinus rhythm (*Figure 3*). She was continued on unchanged treatment remaining in World Health Organization (WHO) FC II 24 months after diagnosis with preserved RV function.

## Patient 2

A 34-year-old woman presented with an 8-month history of reduced exercise capacity and shortness of breath. Her past medical history included a chronic fatigue syndrome and Raynaud’s phenomenon. Cardiorespiratory examination was unremarkable; however, she was noted to have marked exertional desaturation on 6-min walk distance from 97% to 81% achieving a distance of only 75 m. An ECG showed normal sinus rhtyhm (*Figure 3*). An echocardiogram showed a dilated RV with impaired systolic function and a D-shaped left ventricle on short axis view. Lung ventilation perfusion scan with SPECT-LDCT did not show segmental or subsegmental mismatched abnormalities. Computed tomography chest showed widespread subtle thickening of the interlobular septa which was not present 3 years ago. Right heart catheterization demonstrated pre-capillary pulmonary hypertension (*Table 1*, patient 2) and a diagnosis of idiopathic PAH was made with initiation of diuretics and pulmonary vasodilators in a sequential combination strategy.

Approximately 3 years following diagnosis, given mild progression of breathlessness despite up-titration of pulmonary vasodilators and persistent exertional desaturation, she underwent repeat assessment. Echocardiography demonstrated normalization of RV dimension with mildly impaired systolic function and although right heart catheterization supported similar elevation in mean pulmonary artery pressure and pulmonary vascular resistance (PVR) (data not shown). A repeat 6-min walk test distance was 150 m, with desaturation to 87% after only 2 min. High-resolution CT at this point showed interlobular septal thickening and small calcified nodules (*Figure 1B*). Given this marked arterial desaturation, an echocardiogram with bubble injection was arranged which showed moderate right-to-left shunting with late appearance of contrast in left-sided pulmonary veins (*Figure 2B*). Genetics testing demonstrated a homozygous recessive mutation in the EIF2AK4 gene, and her father was found to be heterozygous for EIF2AK4.

The diagnosis was revised to PVOD, and she was started on ambulatory oxygen. Given clinical suspicion of a possible immune-mediated pulmonary vasculopathy, a trial of prednisolone, and mycophenolate mofetil was commenced. Repeat right heart catheterization 2 years later showed an improvement in pulmonary haemodynamics (*Table 1*, Patient 2) with improvement in the degree of interlobular septal thickening on CT. She is presently suspended from the active transplant list and remains clinically stable over 10 years since the first presentation.

## Discussion

We describe two patients with *EIF2AK4*-associated PAH alive 2 and 10 years after diagnosis who both demonstrate an underreported feature potentially unique to their favourable clinical trajectory corroborated by near normalization of pulmonary vascular resistance and preserved WHO functional class following pulmonary vasodilator treatment. Despite typical clinical features of PVOD at presentation (*Table 1*), both patients demonstrated evidence of intrapulmonary shunt (IPS) during resting contrast transthoracic echocardiography (CTTE) undertaken 4 and 36 months into treatment with pulmonary vasodilators. In each case, IPS was presumed to arise from microscopic arteriovenous collateral flow in the lung with no evidence for macroscopic arteriovenous malformations on contrast thoracic CT either at diagnosis or during follow-up. Intrapulmonary shunt increases predisposition to substantial oxygen desaturation on exercise yet, for the hypertensive RV, may also act as a ‘release valve’ facilitating a reduction in RV afterload at the expense of systemic oxygenation. Due to the timing of CTTE in each patient’s clinical course, it is unclear whether IPS predated or developed *in response to* pulmonary vasodilator treatment. However, their trajectory contrasts strikingly with prior reports of development of IPS in classical PAH where the introduction of pulmonary vasodilators has led to a catastrophic fall in systemic oxygenation.^4,5^

The presence of IPS may be inferred from CTTE using injection of agitated saline into a peripheral vein that appears in the left heart within a range between three and five cardiac cycles. This denotes a likely intrapulmonary origin of shunt and differs from an intracardiac communication where earlier contrast appearance appears in the left heart typically between one and three cardiac cycles. Whilst IPS may originate from discrete, microscopic extra-alveolar arteriovenous connections allowing blood to traverse either completely unventilated alveoli or otherwise bypass those with better ventilation, CTTE may also be frequently positive in normal lungs without any detectable impairment in gas exchange.^6–8^ When associated with the former, the hypoxemic effect of extra-alveolar blood flow relies not only upon the size of the shunt but also on the oxygenation saturation of mixed venous blood (SvO_2_), a small shunt with low SvO_2_ giving rise to a more profound hypoxemic effect than a larger shunt with high SvO_2_. Quantification of IPS using the shunt ratio (*Qs/Qt*) may be undertaken using blood gas analysis from the Berggren equation (the ‘shunt equation’) performed with the patient breathing 100% oxygen:


$$\frac{Qs}{Qt}=\frac{Cc_{O2}-Ca_{O2}}{Cc_{O2}-Cv_{O2}}$$

where *Qs* is the shunt flow in litres per minute and *Qt* is the total cardiac output. *Ca_O2_* and *Cv_O2_* correspond to the oxygen content of the systemic arterial and mixed venous blood, respectively, and *Cc_O2_* is the oxygen content of the end-capillary blood (taken as equal to the alveolar partial pressure of oxygen).

In the largest case series of patients with PAH and biallelic *EIF2AK4* mutations, Hadinnapola *et al*.^3^ noted a severe reduction in lung diffusion capacity (Kco 33%), lower resting (SpO_2_ 91%, 90%–94%) and exercise (SpO_2_ 78%, 75%–82%) saturation values, and a reduced likelihood of development of pulmonary oedema in response to pulmonary vasodilator treatment. Survival was also worse in patients with homozygous mutations. In keeping with these findings, both of our patients tolerated the introduction of pulmonary vasodilators without development of pulmonary oedema. In contrast, however, both patients also demonstrated near normalization of PVR on pulmonary vasodilator treatment with notable preservation in exercise saturations on symptom-limited exercise testing.

The favourable clinical course of both patients lead us to postulate that the presence of IPS may confer prognostic advantage in subclasses of patients with PVOD where heterogeneity in venular remodelling allows for persistence of normal capillary channels and bypass of blood around an obstructed venular compartment. Intrapulmonary shunt allows the RV to unload, whilst at the same time permitting passage of highly saturated mixed venous blood especially during exercise with relative preservation in V/Q relationships. A similar mechanism of preserved arterial saturations has been postulated in high cardiac output states such as hepato-pulmonary syndrome where intrapulmonary passage of contrast at rest is frequently associated with minimal hypoxaemia.^9^ Conversely, development of IPS in patients with predominant arteriolar remodelling typical of idiopathic PAH with plexogenic lesions may increase blood flow away from the pulmonary capillary bed via development of *bronchopulmonary* anastomoses, thus increasing V/Q heterogeneity and systemic hypoxaemia.

Our clinical observations in both patients are limited by several unknowns including the lack of lung biopsy data to better characterize the degree of pulmonary venular involvement and a possible effect of immunomodulation used in Patient 2. In line with ESC guidelines on the management of PVOD, however, lung biopsy is rarely recommended. Furthermore, IPS was documented in both cases before initiation of immunomodulatory therapies. In addition, definitive proof of anatomic shunt using blood gas analysis and 100% oxygen study was not undertaken during rest or exercise. Despite these issues, observed recovery of right heart function and the benign clinical trajectory of both patients support a possible beneficial role of IPS in this rare subgroup of PVOD. Future prospective studies employing CTTE in PAH perhaps targeting those with lower diffusion capacity and greater systemic hypoxaemia may offer increased insight into the role of intrapulmonary shunting suggested by CTTE in a condition otherwise associated with a dire prognosis.

## Lead author biography


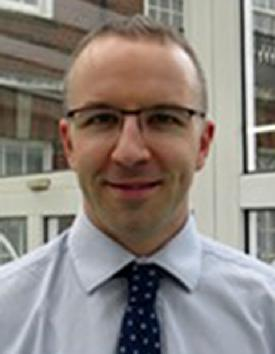


Dr Colm McCabe is a consultant respiratory physician specializing in pulmonary vascular disease and leads the exercise testing services in cardiology at the Royal Brompton Hospital. He is an honorary senior lecturer at Imperial College, London, holding research grants in translational aspects of pulmonary hypertension and right ventricular physiology. His training includes a research MD at Papworth Hospital, Cambridge; clinical fellowship in exercise physiology funded by an award of the Scadding Morriston Davies and Berkeley Fellowships (UCL with Gonville and Caius); and a Masters from the University of Bologna. He has recently co-edited a book on contemporary pulmonary embolism management in hospital practice.

**Consent:** The authors confirm that written consent for submission and publication of this case report including the images and associated text has been obtained from the patient in line with the COPE guidelines.

**Funding:** None declared.

## Data Availability

All clinical data are provided in the written manuscript. There are no extra data or software code upon which the manuscript contents rely.
